# Long-Term Weight Loss Effects of a Behavioral Weight Management Program: Does the Community Food Environment Matter?

**DOI:** 10.3390/ijerph15020211

**Published:** 2018-01-26

**Authors:** Shannon N. Zenk, Elizabeth Tarlov, Coady Wing, Stephen A. Matthews, Hao Tong, Kelly K. Jones, Lisa M. Powell

**Affiliations:** 1 College of Nursing, University of Illinois at Chicago, Chicago, IL 60612, USA; elizabeth.tarlov@va.gov (E.T.); kellykj@uic.edu (K.K.J.); 2 Edward Hines Jr. Veterans Affairs Hospital, Hines, IL 60141, USA; hao.tong@va.gov; 3 School of Public and Environmental Affairs, Indiana University, Bloomington, IN 47405, USA; cwing@indiana.edu; 4 Department of Sociology & Criminology, Pennsylvania State University, University Park, PA 16802, USA; sxm27@psu.edu; 5 Department of Anthropology, Pennsylvania State University, University Park, PA 16802, USA; 6 School of Public Health, University of Illinois at Chicago, IL 60612, USA; powelll@uic.edu

**Keywords:** obesity, weight maintenance, weight loss, weight loss intervention, weight loss maintenance, food store, restaurant, accessibility, neighborhood, food access, MOVE!

## Abstract

This study examined whether community food environments altered the longer-term effects of a nationwide behavioral weight management program on body mass index (BMI). The sample was comprised of 98,871 male weight management program participants and 15,385 female participants, as well as 461,302 and 37,192 inverse propensity-score weighted matched male and female controls. We measured the community food environment by counting the number of supermarkets, convenience stores, and fast food restaurants within a 1-mile radius around each person’s home address. We used difference-in-difference regression models with person and calendar time fixed effects to estimate MOVE! effects over time in sub-populations defined by community food environment attributes. Among men, after an initial decrease in BMI at 6 months, the effect of the program decreased over time, with BMI increasing incrementally at 12 months (0.098 kg/m^2^, *p* < 0.001), 18 months (0.069 kg/m^2^, *p* < 0.001), and 24 months (0.067 kg/m^2^, *p* < 0.001). Among women, the initial effects of the program decreased over time as well. Women had an incremental BMI change of 0.099 kg/m^2^ at 12 months (*p* < 0.05) with non-significant incremental changes at 18 months and 24 months. We found little evidence that these longer-term effects of the weight management program differed depending on the community food environment. Physiological adaptations may overwhelm environmental influences on adherence to behavioral regimens in affecting longer-term weight loss outcomes.

## 1. Introduction

About one-quarter of American adults report that they are “seriously trying to lose weight” [1]. For many of these people, weight loss may have important health benefits [2] because more than two-thirds of adults (69.0%) are overweight or obese, including 71.6% of men and 66.5% of women [3], but it is hard to lose weight. In ideal settings, the treatment effects from behavioral (non-surgical) weight management programs amount to weight loss of about 5–10% after 6 months for the average participant [4,5,6,7,8]. The effects vary across individuals and they tend to fade over time, particularly after about 6 to 9 months [5,9,10,11,12]. Physiological adaptations explain part of the fade out effect: people regain some lost weight as their bodies find an equilibrium that matches new conditions. But longer-term weight loss and maintenance is also hard to achieve because people have trouble adhering to diet and physical activity regimens. Identifying personal and environmental factors that make it easier for people to adhere to a healthy lifestyle may help transform the short-run benefits of weight management programs into longer-term improvements in population health. One possibility is that behavioral weight management programs may have longer lasting effects for people who live in neighborhoods with food environments that facilitate healthy diets.

Considerable research on the community food environment, measured by the number, type, location, and accessibility of food outlets in a community [13], has shown that in the cross-section, people with better geographic access to supermarkets near their home tend to have lower body mass index (BMI) and obesity risk [14,15,16]. Conversely, people with greater geographic access to convenience stores and fast food restaurants tend to have higher BMI and obesity risk [14,15,16]. However, these cross-sectional associations typically weaken or disappear in longitudinal studies that use stronger quasi-experimental research designs to adjust for a broader array of potential confounders [17,18,19,20,21,22,23,24]. Additionally, there is not much research on whether the community food environment alters the treatment effects of behavioral weight management interventions. In particular, there are no studies that examine whether the community food environment might help people maintain the effects of a weight management program over a period of more than one year [25,26,27].

This study used data from the Weight and Veterans’ Environments Study (WAVES) [18] to examine the link between the community food environment and the longer-term effects of the U.S. Department of Veterans Affairs (VA) MOVE! program, which is a large-scale behavioral weight management program, addressing both diet and physical activity behaviors, that operates in facilities across the country. In a prior WAVES analysis, we found that male MOVE! participants with greater access to convenience stores within one mile lost less weight at 6-month relative to controls [27]. Our central hypothesis for the analysis in this study was that the short-term treatment effects of the intervention would survive longer for people living in more supportive community food environments. To test this hypothesis, we estimated the treatment effect of the MOVE! program over 18 months at 6-month intervals beginning at the end of the initial 6 months of the program and ending at 24 months. We compared these time-varying treatment effects for sub-populations of people with different geographic access to supermarkets, convenience stores, and fast food restaurants. 

## 2. Materials and Methods

### 2.1. Data

Patient-level data came from the 2009–2014 VA Corporate Data Warehouse and VA/CMS data repository, which are repositories of patient-level clinical and administrative data from the electronic health record and other sources. These data were geocoded and linked with secondary environmental data from Dun and Bradstreet (Chicago, IL, USA), InfoUSA (Papillion, NE, USA), and other sources. 

### 2.2. Intervention

The VA MOVE! program is a weight management program for military veterans receiving VA healthcare that is available in VA medical centers and community-based outpatient centers across the country. VA MOVE! was implemented nationwide in 2006. It is modeled on an updated version of Diabetes Prevention Program, which is a lifestyle intervention that was successful in clinical trials [28,29,30,31,32]. VA clinical guidelines recommend that providers refer patients who are obese or who are overweight and also have obesity-related comorbidities and no contraindications to weight loss [33]. MOVE! participants receive an individualized treatment plan, including education and counseling to support diet and physical activity behavior change. The program offers group and individual sessions, and it also allows people to participate remotely through in-home or clinical video telehealth modalities.

### 2.3. Research Design

We used a two-stage research design. In the first stage, we developed inverse propensity score weights to build a matched control group of non-participants who closely resembled MOVE! participants on a rich set of baseline covariates. In the second stage, we used generalized difference in difference (DID) regressions to control for additional sources of bias and to estimate treatment effects at different times and in different sub-populations. Using a matched control group minimizes selection bias that might arise if MOVE! participants and non-participants differ on demographic, clinical, and environmental factors at baseline. The DID regressions control for any remaining unmeasured and time-invariant confounders that differ across the two groups. It also controls for unmeasured and time-varying confounders that affect both groups in the same way. The study was approved by the institutional review boards of the University of Illinois at Chicago (2013-0650) and Edward Hines, Jr. VA Hospital (13-043).

### 2.4. Sample

The analytic sample consisted of 560,173 male veterans and 52,577 female veterans 20–80 years of age at baseline who received primary healthcare services in the VA 2009–2014 and lived in metropolitan counties of the continental U.S. The cohort from which the sample is drawn excludes people without at least one VA healthcare encounter in the two years prior to baseline; with long-stay nursing home residence at baseline; without at least one home address geocoded to the street or ZIP + 4 level; and without valid and clinically plausible height (at least one) and weight (at least two) measurements [18,27]. For this analysis, we defined MOVE! participants as people who had at least two MOVE! visits within a 6-month period, no MOVE! visits within the 12 months prior to the initial MOVE! visit (baseline), and had a weight measurement at least at baseline and either 18 or 24 months follow up. By this definition, 98,871 men and 15,385 women in the analytic sample were MOVE! participants. From the remainder of the sample of non-participants, we derived a control group roughly five times the size of the MOVE! sample (i.e., five controls per MOVE! participant): 461,302 men and 37,192 women. For the control group, baseline was the date on which the first body weight measurement in a 6-month “intervention” period was taken. However, because multiple 6-month “intervention” periods were available for many non-participants and to make sure that these periods were distributed over the 2009–2014 study period in the same manner as the MOVE! group, we randomly selected control group baseline dates with probability proportional to the relative frequency of baseline dates in the MOVE! group.

### 2.5. Propensity Score Matching Procedure

We used propensity score analysis to match the control group to the MOVE! group on baseline covariates. We estimated a logistic regression model of MOVE! participation on a set of 120+ baseline covariates including veteran demographics, clinical factors, healthcare utilization, residential environmental attributes, and VA healthcare facility characteristics. The estimated propensity scores were predicted probabilities from the model. To form the matched sample, we assigned each control a weight equal to $\hat{p_{i}}/\left(1-\hat{p_{i}}\right)$ and each participant a weight of 1. In this sense, the control group sample was weighted to match the distribution of covariates in the participant sample.

We used an iterative procedure to choose an appropriate specification for the propensity score model. We started by constructing the weights implied by a simple candidate model specification. Then we assessed covariate balance in the weighted sample. When one or more covariates had a standardized difference in excess of 0.05 standard deviations, we re-specified the candidate model to incorporate more non-linearity with respect to the out of balance covariates. We continued the process until all covariates had standardized differences of less than 0.05 standard deviations, indicating an excellent match between the intervention and control groups. More information on the propensity score analysis is provided elsewhere [18,27].

### 2.6. Measures

#### 2.6.1. Body Mass Index (BMI)

We calculated BMI (weight in kg/height in m^2^) at baseline and at four follow-up points (6-, 12-, 18- and 24-month after baseline), based on weight and height measurements taken during clinical encounters. To reduce measurement error, we set each person’s height to the modal value of his or her measurements during the study period [17]. Ideally, for MOVE! participants, baseline body weight would have been measured on the date of the initial MOVE! visit and the 6-, 12-, 18-, and 24-month follow-up body weights would have been measured at 180, 360, 540, and 720 days after baseline. However, the body weight data come from clinical encounters and so we usually did not have a weight measurement on the exact target day. Thus, for each person, we defined a window at the baseline date (±30 days) and each follow-up target (±90 days). We used the weight measurement within the person’s window that occurred the closest to the target day. Our decision to use a smaller window at baseline was based on the need for a weight as close to the date of MOVE! initiation as possible. For the control group, baseline was the date on which the first weight measurement in a 6-month “intervention” period was taken and the follow-up weights were selected in the same way as for MOVE! participants.

#### 2.6.2. MOVE! Exposure

Clinic stop codes, an identifier assigned by the VA Managerial Cost Accounting Office that defines the clinical service a patient received, were used to identify individual, group, and telephone MOVE! visits. We used a dichotomous indicator for MOVE! participation (1 = MOVE participant; 0 = control).

#### 2.6.3. Community Food Environment Exposures

We obtained yearly home address geocodes, up to date as of the end of each VA fiscal year (30 September), from the VHA Planning Systems Support Group. All included observations were geocoded to a specificity of street address, centroid of street segment, or centroid of the ZIP + 4 area. We constructed annual environmental measures based on centroids of a 30 m × 30 m grid (approximately 9 billion grid cells) across the continental U.S. and assigned values for the environmental measures to individuals for each year (i.e., annual measures) according to the cell in which his or her home geocode was located during that year. We computed the number of supermarkets, convenience stores, and fast food restaurants within a 1-mile Euclidean radius of the person’s residence. We selected one mile because not only is it the most commonly used distance to measure the community food environment [16] but also to capture the immediate vicinity of people’s home, which may be especially relevant for convenience stores because people tend to frequent these types of outlets closer to home [34]. As mentioned below (Section 2.8), we also tested the sensitivity of results to a 3-mile radius (given that people typically shop for groceries 2–4 miles from their home) [35,36,37,38,39,40] and a 0.5-mile radius (as a measure of walking distance), as well as proximity measures (distance to the nearest food outlet by type). Food outlet counts or densities (per area) are the most commonly used measures of the community food environment [15,16,41]. The supermarket (standard industrial classification (SIC) codes 541101, 541102, 541104–541109 and >$2 million annual sales) and convenience store (SIC code 541103, 554101, 554103) data came from InfoUSA. The fast food restaurant (SIC code 58120601, 58120602, 581203 but not coffee shops: 58120304) data came from Dun & Bradstreet. Baseline and 6-month BMI measures were linked to the person’s year 1 environmental data, 12- and 18-month BMI to year 2 environmental data, and 24-month BMI to year 3 environmental data. More information on the community food environment measures can be found elsewhere [17,18,42].

#### 2.6.4. Covariates

DID regression models included the following time-varying individual-level covariates: marital status (married, separated or divorced, widowed, single, unknown) and ten chronic health conditions associated with both BMI and independently with diet and/or physical activity (breast cancer, cerebrovascular disease, colon cancer, congestive heart failure, depression, diabetes, hyperlipidemia, hypertension, myocardial infarction, and osteoarthritis). The models also included time-varying area-level covariates: census division (New England, Middle Atlantic, East North Central, West North Central, South Atlantic, East South Central, West South Central, Mountain, Pacific) and urbanicity at the county level (large central metro, large fringe metro, medium metro, and small metro) [43]. National Center for Health Statistics (NCHS) county classification codes for urbanicity were available for 2006 and 2013 only; thus, we assigned 2006 NCHS codes for urbanicity to individuals’ residential location for years 2009–2012 and 2013 NCHS codes to individuals’ residential location for years 2013–2014. We also adjusted for census tract SES (poverty rate and median household income, both categorized into deciles of the distribution of values for all continental U.S. census tracts) and population density (quartiles of the distribution of values for all continental U.S. census tracts) using information from the American Community Survey 5-year estimates. In addition, we controlled for counts of the number of grocery stores (InfoUSA), parks (NAVTEQ, Amsterdam, The Netherlands and TeleAtlas, Amsterdam, The Netherlands), and fitness facilities (InfoUSA) within 1-mile. We also controlled for month and year of weight measurements (to account for seasonality), the number of days from each target follow-up date and the actual weight measurement date, VA facility where the individual was most frequently seen, and distance to the nearest VA facilities for outpatient (hospital-based or community-based) and inpatient care. Four dummy variables indicated whether the observation was baseline versus 6-, 12-, 18-, or 24-month follow-up. The propensity score model controlled for baseline values of all of the variables included in the DID regression models and also adjusted for several more time-invariant factors. A full list of the propensity score model variables is provided elsewhere [18].

### 2.7. Statistical Model

The basic research design in our study operates under the assumption that, in the absence of the MOVE! program, average BMI would have followed the same trajectory over time in the treatment group and the matched control group. We invoke the same common trend assumption in sub-populations defined by measures of the food environment. We operationalize the research design by estimating regression models that allow treatment effects to vary across sub-populations using interaction terms. In the most basic specification, treatment effects are restricted to be constant over time:

(1) $$BMI_{it}=X_{it}\alpha +\beta \left(Move_{i}\times Post6_{it}\right)+\delta \left(Move_{i}\times Post6_{t}\times Food_{it}\right)+\epsilon_{it.}$$

In the model, $Food_{it}$ is a vector of food outlet covariates, $Post6_{t}$ is a binary variable set to one for all observations taken 6 or more months after the person’s baseline period, and $Move_{i}$ is a binary variable set to one if the person is a MOVE! participant. $X_{it}$ is a vector that includes covariates and main effects. β represents the DID estimate of the treatment effect in the reference group and δ is a vector of coefficients that measures the difference in the treatment effect associated with a particular food outlet access sub-population. To find the treatment effect in any particular food environment sub-population, add the relevant δ to the reference group treatment effect. All of the regression coefficients were estimated using inverse propensity score weights to create the matched sample of controls. We also examined augmented versions of the model that include person and calendar time fixed effects.

To study the way that treatment effects persist or fade out over time, we expanded the basic specification to allow treatment effects that change over time. The time-varying treatment effect model that we work with is:

(2) $$\begin{array}{cc} BMI_{it}=X_{it}\alpha + & \sum_{m\in \left\{6,12,18,24\right\}}[\beta_{m}\left(Move_{i}\times Post{\left(m\right)}_{it}\right)\\ & +\delta_{m}\left(Move_{i}\times Post{\left(m\right)}_{it}\times Food_{it}\right)]+\epsilon_{it} \end{array}$$

In the time-varying model, $Post{\left(m\right)}_{it}$ is a dummy variable set to one for all observations recorded m or more months after the person’s baseline period for values of m={6,12,18,24}. $\beta_{6}$ is the DID estimate of the treatment effect in the community food environment reference group at 6 months following baseline. The effect at 12 months is $\beta_{6}+\beta_{12}$; the effect at 18 months is $\beta_{6}+\beta_{12}+\beta_{18};$ and the effect at 24 months is $\beta_{6}+\beta_{12}+\beta_{18}+\beta_{24}$. Adding in the relevant $\delta_{m}$ estimates makes it possible to compute period specific treatment effects in sub-populations with different food environments. We also examined augmented versions of the time-varying treatment effects specification that allowed for person and calendar time fixed effects.

As described in more detail elsewhere [17,18,27], the models outlined above account for a broad set of unmeasured, time-invariant confounders and unmeasured, time-varying confounders that affect the MOVE! and control groups. However, these models may still be biased by other unmeasured, time-varying factors that are correlated with both BMI and food outlet access. For instance, if changes in obesity relevant lifestyle preferences that influence where a person chooses to live are different among MOVE! participants compared to controls, DID estimates of MOVE! treatment effects may be biased. Specifically, the DID estimate may make it appear that a change in supermarket access between two time points is associated with less weight gain over time when the change was actually due to the change in lifestyle preference that precipitated the change in supermarket access. To address such concerns, we also estimated models in a limited sample of MOVE! participants and controls who remained within 0.25 mile of their home location at baseline throughout the study period (“stayers”). Among stayers, the DID models identify treatment effects using food outlet openings and food outlet closures.

All models were estimated using the inverse propensity score weights and standard error estimates allowed for clustering of individuals within counties at baseline using a Huber-White cluster robust variance matrix. Analyses were conducted separately for men and women because men comprised over 90% of the sample, and they differ in their demographic profiles with women as a group being younger with more non-Hispanic blacks. We used Stata version 14 (StataCorp LLC, College Station, TX, USA) for all analyses.

### 2.8. Sensitivity Analyses

We conducted a variety of sensitivity analyses to check the robustness of our results. First, we conducted the analyses in alternative samples: (a) those with body weight measurements at baseline and all follow-up points (6, 12, 18, and 24 months) and (b) those with body weight measurements at a minimum at baseline and 18 months. Second, we tried different operational definitions of our key independent variables: a combined supermarket and grocery store variable (rather than as two separate variables), “large” chain supermarkets (those with >50 employees and part of a national chain), chain and non-chain fast food restaurants as separate variables (rather than as a combined variable), relative accessibility of supermarkets to fast food restaurants, per capita counts of each outlet type (supermarket, convenience store, fast food restaurant), counts of each food outlet type within 3 miles, presence of each food outlet type within 0.5 mile (as a measure of walking distance), and distance to the nearest outlet by food outlet type. Overall, results of these analyses did not differ meaningfully from what is presented below (see Supplementary Materials).

## 3. Results

### 3.1. Descriptive Statistics

Table 1 shows propensity-score weighted descriptive statistics for the MOVE! group and the control group, separately for men (*n* = 98,871 MOVE! participants and *n* = 461,302 controls) and women (*n* = 15,385 MOVE! participants and *n* = 37,192 controls). Mean BMI in the MOVE! group was 36.09 for men and 35.43 for women, and most (84.09% of men, 80.03% of women) were obese. The control group is very similar to the MOVE! group on all the weighted sample characteristics, which shows that the propensity score model was successful in creating a covariate balanced control group (Table 1).

### 3.2. Average MOVE! Effect on Longer-Term BMI Change at 12, 18, and 24 Months Following Initial 6-Month BMI Change

**Figure 1:**
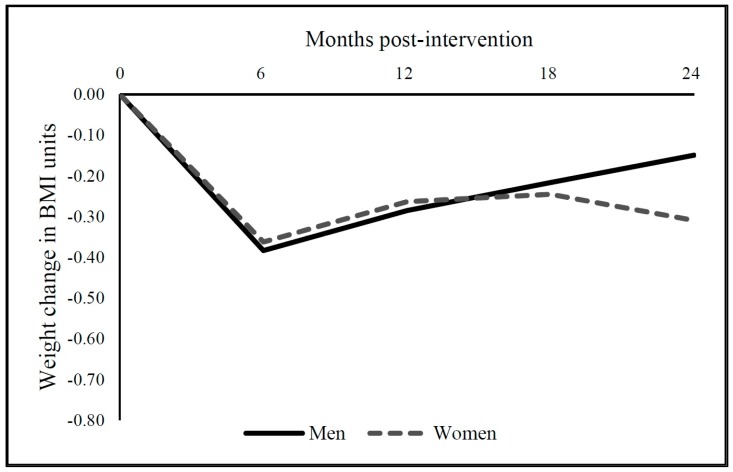
MOVE! effect on initial 6-month BMI change and on longer-term BMI change at 12, 18, and 24 months, separately for men and women. Statistically significant initial MOVE! effect at 6 months for men (−0.383, *p* < 0.001) and women (−0.362, *p* < 0.001) and incremental changes at 12 months for men (0.098, *p* < 0.001) and women (0.099, *p* < 0.05); and for men at 18 months (0.069, *p* < 0.001) and at 24 months (0.067, *p* < 0.001). These are difference-in-difference estimates of the effect of MOVE! on BMI change from ordinary least squares (OLS) regression models with person and calendar time fixed effects and the following covariates: marital status, 10 chronic health conditions, census tract median household income, census tract poverty rate, census tract population density, census division, urbanicity, VA facility, distance to the nearest VA facilities for outpatient and inpatient care, number of days from each target follow-up date and the actual weight measurement date, supermarkets, convenience stores, fast food restaurants, grocery stores, parks, and fitness facilities.

Figure 1 shows DID results for the average MOVE! effect on initial 6-month BMI change and on longer-term BMI change at 12, 18, and 24 months, adjusting for food outlet access and individual and area covariates. In men, MOVE! participation was associated with an average −0.383 kg/m^2^ change in BMI at 6 months relative to controls (*p* < 0.001). Between 6 months and 12 months, male MOVE! participants had an average BMI increase of 0.098 kg/m^2^ (*p* < 0.001). In the following two 6-month periods, men also increased their BMI incrementally by 0.069 kg/m^2^ at 18 months (*p* < 0.001), and 0.067 kg/m^2^ at 24 months (*p* < 0.001), on average.

In women, MOVE! participation was associated with an average −0.362 kg/m^2^ change in BMI at 6 months relative to controls (*p* < 0.001), but an average 0.099 kg/m^2^ incremental increase at 12 months (*p* < 0.05) and non-significant incremental changes at 18 months (b = 0.018, *p* > 0.05) and 24 months (b = −0.064, *p* > 0.05).

### 3.3. MOVE! Effect on Longer-Term BMI Change at 12, 18, and 24 Months for Sub-Populations with Different Food Environments

Table 2 shows DID results for the incremental effect of MOVE! on longer-term BMI change at 12, 18, and 24 months (with the initial 6-month BMI change shown in the left column in each panel) for sub-populations with different food environments, adjusting for individual and area covariates. Statistically significant effects indicate a difference in BMI change in that group relative to those with no food outlets within one mile. In men, between 12 and 18 months, BMI change was different in MOVE! participants with no food outlets within 1-mile and those with 1–2 convenience stores within 1-mile; those with 1–2 convenience stores had a greater BMI increase (b = 0.078, *p* < 0.05). Between 18 and 24 months, there was a difference in BMI change between MOVE! participants with no food outlets within 1-mile and two groups: MOVE! participants with 1–2 convenience stores within 1-mile and MOVE! participants with 1–4 fast food restaurants within 1-mile. Compared to MOVE! participants with no outlets, MOVE! participants with 1–2 convenience stores had a decrease in BMI (b = −0.094, *p* < 0.05). Compared to MOVE! participants with no outlets, MOVE! participants! with 1–4 fast food restaurants had an increase in BMI (b = 0.103, *p* < 0.05). There were no statistically significant associations between MOVE! participation and BMI change at 12, 18, or 24 months by supermarket access in men.

Table 2 also shows associations for women. In women, the MOVE! effect on BMI change at 12, 18, or 24 months did not differ across sub-populations with different access to food outlets near home.

**Figure 2:**
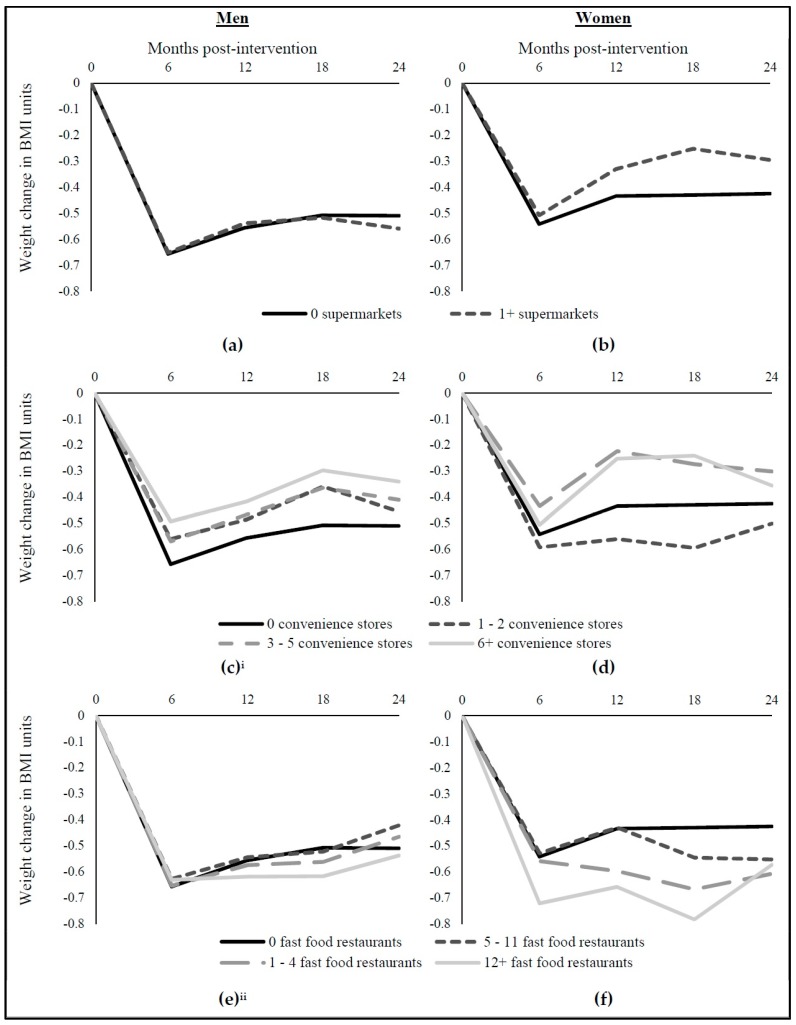
MOVE! effect on 12-, 18-, and 24-month BMI Change (as well as on Initial 6-month BMI Change) by Supermarket Access for (**a**) Men and (**b**) Women; Convenience Store Access for (**c**) Men and (**d**) Women; and Fast Food Restaurant Access for (**e**) Men and (**f**) Women. ^i^: Statistically significant difference in MOVE! effect between men with no food outlets and men with low (1–2 stores) convenience store access at 18 months (*p* < 0.05) and 24 months (*p* < 0.05) and at 6 months for men with low (*p* < 0.01), medium (3–5 stores) (*p* < 0.05) and high (6 + stores) (*p* < 0.001) convenience store access; ^ii^: Statistically significant difference in MOVE! effect for men with low (1–4 restaurants) fast food restaurant access (*p* < 0.05) at 24 months.

Figure 2 illustrates these effects of MOVE! on BMI change at 6, 12, 18, and 24 months for both men and women with different access to supermarkets, convenience stores, and fast food restaurants, respectively.

### 3.4. MOVE! Effect on Longer-Term BMI Change at 12, 18, and 24 Months for Sub-Populations with Different Food Environments among Stayers

Table 3 shows results for stayers only (82.2% of men and 75.0% of women), or those whose homes remained within 0.25 mile of their location at baseline throughout the study period. Overall, patterns of results were similar. Contrary to our hypothesis, between 6 and 12 months, BMI change was different in male MOVE! participants with no food outlets within 1-mile and those with 12 or more fast food restaurants. Those with no food outlets increased their BMI while those with 12 or more fast food restaurants decreased their BMI (b = −0.154, *p* < 0.05). Between 18 and 24 months, BMI change was different in male MOVE! participants with no food outlets within 1-mile and those with 1–4 fast food restaurants. Those with no food outlets had no change in BMI while those with 1-4 fast food restaurants increased their BMI (b = 0.103, *p* < 0.05). None of the other differences in BMI change between the sub-population with no food outlets and the sub-populations with food outlets were statistically significant.

Among women, only one difference was statistically significant. Between 18 and 24 months, BMI change was different in female MOVE! participants with no food outlets within 1-mile and those with 1–4 fast food restaurants; those with 1–4 fast food restaurants had a greater BMI decrease (b = −0.330, *p* < 0.05) (Table 3).

## 4. Discussion

To our knowledge, this was the first study to test whether the community food environment helps people maintain the treatment effects generated by behavioral weight management programs over a period longer than 6–12 months. Building on our prior study on the effect of MOVE! on initial 6-month weight loss [27], we found that, on average, male MOVE! participants began regaining weight by 12 months after initiation of the MOVE! program, increasing their BMI, on average, by an additional 0.098 kg/m^2^ (25.6% of their initial 6-month −0.383 kg/m^2^ change in BMI), 0.069 kg/m^2^, and 0.067 kg/m^2^ at 12, 18, and 24 months, respectively. Overall, female MOVE! participants regained weight (0.099 BMI units, or 27.3% of their initial 6-month −0.362 unit change in BMI) by 12 months, but then their weights stabilized. We found little evidence that longer-term success at 12, 18, or 24 months significantly differed depending on the community food environment near home, specifically access to supermarkets, convenience stores, or fast food restaurants.

Our results suggest that the community food environment close to home is not an important contributor to longer-term weight loss outcomes nor is it a significant factor in individual variability in longer-term weight loss. As a group, MOVE! participants were able to maintain a relatively large portion of their initial weight loss regardless of their access to outlets that predominately sell energy-dense, nutrient poor foods (convenience stores, fast food restaurants) near their home. Our prior work suggested that male MOVE! participants with more convenience stores—which predominately sell energy-dense, nutrient poor food products—within one mile of their home lost less weight at 6 months [27]. Thus, it is possible that the residential food environment plays a larger role in men’s ability to lose weight initially but is less consequential in weight loss maintenance. One potential explanation is that the ease and speed at which men can obtain energy-dense food products at convenience stores near home is tempting when they are initially trying to lose weight but becomes less enticing later after they gain new self-regulation skills and alter their food procurement patterns. It is also possible that physiological adaptation and other dynamics that are common following the initial weight loss period overwhelm environmental influences.

Our work extends prior studies focused on the role of the community and/or consumer food environment in the effectiveness of weight loss or dietary interventions. Fiechtner et al., studied an obesity intervention in a sample of 498 children in Massachusetts [26]. At a one-year follow up, they found that the intervention led to more weight loss for children living closer to supermarkets. Specifically, living 1 mile closer to a supermarket reduced BMI by 0.04 standard deviations relative to controls. They also found that living 1 mile closer to a supermarket was associated with a 0.29 serving increase in fruit and vegetable intake relative to controls, but was not associated with change in sugar-sweetened beverage intake relative to controls. Using a one-group pre-post design, Mendez et al., examined 127 adults participating in a weight loss intervention [25]. They found no association between grocery store density or restaurant density and weight change at 6 months. Wedick et al., conducted a randomized control trial of 240 adults with metabolic syndrome in Massachusetts testing the efficacy of two dietary change interventions [44]. Across the two groups when examining “peak” (maximal) dietary change over 12 months, they found that living at least 20 miles from the nearest store with “adequate” availability of healthy food (at or above the sample median) was associated less improvement in daily fiber intake (−1.07 g) and fruit and vegetable intake (−0.19 servings), but not in whole grain intake or overall dietary quality. Effects did not differ by intervention group. Interestingly when examining effects at specific time points (3, 6, and 12 months), they found living at least 20 miles from the nearest store with adequate availability of healthy foods was associated with less improvement in fruit and vegetable intake but they found no other effects at 3, 6, or 12 months. Finally, Gustafson et al., conducted a randomized control trial to test the effect of a behavioral weight loss intervention in 156 low-income women in North Carolina [45]. They examined effects of the food environment on change in fruit and vegetable intake over 16 weeks. Intervention participants who lived in a census tract with a low density of supermarkets had a 1.62 serving increase in fruit and vegetable intake relative to controls. Also, those who perceived their primary food store as low in healthy foods and in low-fat foods had greater increases in fruit and vegetable intake relative to controls. Several other measures of the food environment (e.g., perceived neighborhood healthy food availability, objectively measured food availability at primary store, perceived healthy food affordability at primary store) were not associated with change in fruit and vegetable intake. Differences in the samples and methodologies make it difficult to compare results across studies. Moreover, none of these studies examined effects beyond one year. However, it is noteworthy that Wedick et al., also found that the effect of the food environment on outcomes faded over time [44], which we observed in our study, too.

Important strengths of this study are its relatively long 24-month follow-up, large samples of 560,173 men and 52,577 women that provide sufficient statistical power to detect small effects of the community food environment, and nationwide coverage with considerable diversity in the environment. These features are rare in weight loss interventions. Our study also relied on a strong quasi-experimental research design, which used propensity score analysis to form a matched control group and a multiple period generalized DID design to account for a large class of possible confounders that may have escaped the matching procedure. We also conducted a wide range of sensitivity analyses which demonstrated the robustness of our results to alternative samples and operational definitions of the community food environment.

Nonetheless, our study has several limitations. First, our focus was on the community food environment only; we did not have direct measures of the consumer food environment such as the availability, price, quality, or promotion of healthy and unhealthy foods at food outlets near home [13]. While food outlet type is a reasonable proxy and supermarkets on average carry more healthy foods than grocery stores and convenience stores, healthy and unhealthy food availability do vary among food outlets of the same type [46,47,48,49]. Future research is needed that goes beyond the community food environment in order to better understand the potential role of the food environment in outcomes of weight loss interventions. Second, individual measures of socioeconomic status are not available in our dataset and thus residual confounding is possible if MOVE! participants and non-participants tended to experience systematically different pattern of socio-economic changes over time. Third, the external validity of our study is unclear because the VA population tends to be lower income with disproportionately more non-Hispanic blacks as compared to the U.S. adult population, especially among female veterans [18]. Uncertainty about external validity is a common problem in weight loss interventions because very few have representative samples. Finally, given we characterized the environment for the entire country for seven years based on a 30 m × 30 m raster, we used Euclidean distance rather than street network distance buffers. Therefore, the distances may overestimate food outlet access and be less accurate for people traveling over the street network in areas without a grid street pattern, but may accommodate non-street travel routes including off-street pedestrian pathways and train routes.

## 5. Conclusions

In conclusion, in this study of the VA MOVE! weight management program, we sought to understand whether the community food environment might help to explain poor long-term weight loss outcomes following initial weight loss through lifestyle interventions, and considerable individual heterogeneity in these outcomes. As with prior lifestyle interventions [9], we found that while they were successful in losing weight by 6 months (i.e., reducing BMI relative to matched controls), MOVE! participants had regained weight by 12 months after the initiation of the MOVE! program and the community food environment did not explain individual differences in long-run weight outcomes at 12, 18, or 24 months post-intervention. Still, the role of the environment is an underexplored area of research to understand and potentially improve weight loss interventions that could have important implications for the design of more effective interventions.

## Figures and Tables

**Table 1:** Propensity score weighted descriptive statistics of sample characteristics at baseline for MOVE! participants and controls.

Characteristic	Men		Women	
	MOVE! Participants	Controls	MOVE! Participants	Controls
Total N	98,871	461,302	15,385	37,192
Body Mass Index in kg/m^2^, Mean (Standard Deviation)				
Body Mass Index (BMI)	36.09 (6.62)	36.18 (6.6)	35.43 (6.45)	35.51 (6.35)
Body Weight Status, %				
Normal/Underweight	1.42	1.39	1.81	1.76
Overweight	14.49	14.15	18.15	17.65
Obese	84.09	84.46	80.03	80.58
Age, Mean (Standard Deviation)	59.99 (9.84)	60.20 (9.73)	50.11 (10.76)	50.19 (10.73)
Race, %				
Non-Hispanic White	63.07	62.84	49.24	49.17
Non-Hispanic Black	24.14	24.35	38.38	38.63
Non-Hispanic Other	2.13	2.14	2.90	2.95
Hispanic	5.61	5.68	4.59	4.53
Unknown	5.04	5.00	4.90	4.73
Marital Status, %				
Married	54.99	54.94	30.84	30.62
Separated/Divorced	25.19	25.16	37.32	37.55
Widowed	3.21	3.23	3.85	3.74
Single	16.14	16.19	27.34	27.45
Unknown	0.47	0.48	0.65	0.63
Health Status, %				
Breast Cancer	0.04	0.06	2.53	2.46
Colon Cancer	0.81	0.82	0.31	0.27
Cerebrovascular Disease	6.02	6.07	3.19	3.29
Congestive Heart Failure	7.99	8.24	2.07	2.05
Depression	36.19	36.6	53.24	53.53
Diabetes	45.18	45.98	21.47	21.5
Hyperlipidemia	63.87	64.84	41.31	41.48
Hypertension	73.81	74.68	45.98	46.22
Myocardial Infarction	3.78	3.86	0.81	0.85
Osteoarthritis	22.57	23.58	20.06	21.25
Census Tract Characteristics, Mean (SD)				
Population Density (1000 people/mi^2^)	4.76 (10.15)	4.77 (9.96)	4.42 (8.60)	4.46 (8.79)
% Below Poverty	15.41 (11.96)	15.48 (12.03)	16.12 (11.66)	16.21 (11.66)
Median Household Income	52,911 (21,868)	52,847 (21,854)	51,309 (20,339)	51,194 (20,238)
Urbanicity, %				
Large Central Metro	32.01	32.08	32.05	32.19
Large Fringe Metro	22.83	22.57	21.47	21.48
Medium Metro	30.02	29.89	32.59	32.35
Small Metro	15.14	15.46	13.89	13.99
Census Division, %				
New England	4.60	4.71	2.63	2.65
Middle Atlantic	10.83	10.76	8.03	8.06
East North Central	17.77	18.23	14.23	15.04
West North Central	6.50	6.35	6.29	5.54
South Atlantic Delaware	21.06	21.02	26.37	26.56
East South Central	4.91	4.59	6.38	6.19
West South Central	12.30	11.67	15.41	14.97
Mountain Arizona	10.58	11.07	10.17	10.65
Pacific Alaska	11.44	11.59	10.50	10.36
Supermarkets **^a^**, %				
0 Store	44.79	44.33	44.37	44.29
1 + Store	55.21	55.67	55.63	55.71
Convenience Stores **^b^**, %				
0 Store	21.53	21.18	18.75	18.71
1–2 Stores	22.50	22.51	23.17	23.58
3–5 Stores	25.50	25.71	27.75	26.92
6 + Stores	30.48	30.61	30.33	30.79
Fast Food Restaurants **^b^**, %				
0 Restaurant	18.81	18.57	17.56	17.88
1–4 Restaurants	25.17	24.65	26.07	25.54
5–11 Restaurants	26.97	27.53	28.20	27.71
12 + Restaurants	29.06	29.25	28.16	28.88
Grocery Stores **^a^**, %				
0 Store	49.83	49.44	49.48	49.17
1 + Stores	50.17	50.56	50.52	50.83
Parks **^b^**, %				
0 Park	29.28	28.88	30.95	30.18
1 Park	17.55	17.31	18.23	18.77
2–3 Parks	24.53	24.85	24.45	24.48
4 + Parks	28.65	28.96	26.37	26.56
Fitness Facilities **^b^**, %				
0 Facility	25.90	25.58	25.54	25.32
1–2 Facilities	27.97	28.14	29.94	29.99
3–4 Facilities	18.20	18.29	18.44	18.33
5 + Facilities	27.93	28.00	26.08	26.36

**^a^** For environmental settings for which less than 50% of the full WAVES cohort had a setting within 1 mile (supermarkets, grocery stores), we used a binary variable (0, 1 or more). **^b^** For environmental settings for which at least 10% of the full WAVES cohort had no setting within 1 mile (fast food restaurants, convenience stores, parks, and fitness facilities), we used a 4-category variable, constructed as 0 and then tertiles of the non-zero distribution of values.

**Table 2:** MOVE! effect on longer-term incremental BMI change at 12, 18, and 24 months (mo) by food outlet access.

	Men				Women				
Food Outlet Access	6 mo	12 mo	18 mo	24 mo	6 mo	12 mo	18 mo		24 mo
	b (SE)	b (SE)	b (SE)	b (SE)	b (SE)	b (SE)	b (SE)		b (SE)
MOVE! Effect **^a,b^**	−0.657 ***	0.101 ***	0.048	−0.002	−0.542 ***	0.108	0.004		0.005
	(0.025)	(0.027)	(0.026)	(0.031)	(0.070)	(0.079)	(0.073)		(0.094)
Supermarkets **^a,c^**									
1 + Store	0.006	0.012	−0.027	−0.040	0.034	0.070	0.074		−0.048
	(0.025)	(0.030)	(0.024)	(0.034)	(0.060)	(0.082)	(0.072)		(0.104)
Fast Food Restaurants **^a,c^**									
1–4 Restaurants	0.030	−0.018	−0.026	0.103 *	0.014	−0.009	−0.120	−0.012	
	(0.033)	(0.039)	(0.033)	(0.041)	(0.085)	(0.100)	(0.098)	(0.126)	
5–11 Restaurants	0.004	−0.022	−0.036	0.099	−0.016	−0.147	−0.074	0.055	
	(0.042)	(0.050)	(0.038)	(0.053)	(0.108)	(0.149)	(0.123)	(0.160)	
12 + Restaurants	0.026	−0.089	−0.046	0.081	−0.179	−0.045	−0.129	0.205	
	(0.049)	(0.054)	(0.044)	(0.061)	(0.124)	(0.154)	(0.156)	(0.181)	
Convenience Stores **^a,c^**									
1–2 Stores	0.097 **	−0.026	0.078 *	−0.094 *	−0.050	−0.076	−0.038	0.088	
	(0.031)	(0.036)	(0.032)	(0.042)	(0.084)	(0.116)	(0.108)	(0.145)	
3–5 Stores	0.088 *	0.001	0.055	−0.044	0.108	0.103	−0.054	−0.033	
	(0.037)	(0.042)	(0.038)	(0.048)	(0.103)	(0.141)	(0.120)	(0.145)	
6 + Stores	0.164 ***	−0.025	0.072	−0.041	0.036	0.146	0.007	−0.120	
	(0.040)	(0.045)	(0.040)	(0.055)	(0.134)	(0.181)	(0.132)	(0.168)	

**^a^** Difference-in-difference estimate (b) with standard error (SE) of the effect of MOVE! on BMI change from OLS regression models with person and calendar time fixed effects and the following covariates: marital status, 10 chronic health conditions, census tract median household income, census tract poverty rate, census tract population density, census division, urbanicity, VA facility, distance to the nearest VA facilities for outpatient and inpatient care, number of days from each target follow-up date and the actual weight measurement date, grocery stores, parks, and fitness facilities; **^b^** MOVE! effect for those with no supermarket, fast food restaurant, or convenience store within a 1-mile buffer; **^c^** Difference in MOVE! effect on BMI change relative to those with no food outlets. * *p* < 0.05; ** *p* < 0.01; *** *p* < 0.001

**Table 3:** MOVE! effect on longer-term incremental BMI change at 12, 18, and 24 months (mo) by food outlet access, “stayers” only.

	Stayers								
	Men (*n* = 460,466)					Women (*n* = 39,426)			
Food Outlet Access	6 mo	12 mo	18 mo	24 mo		6 mo	12 mo	18 mo	24 mo
	b (SE)	b (SE)	b (SE)		b (SE)	b (SE)	b (SE)	b (SE)	b (SE)
MOVE! Effect **^a,b^**	−0.507 ***	0.129 ***	0.086 **		0.011	−0.413 ***	0.314 *	0.012	0.140
	(0.025)	(0.030)	(0.029)		(0.036)	(0.097)	(0.122)	(0.102)	(0.109)
Supermarkets **^a,c^**									
1 + Store	0.008	0.031	−0.007	−0.008		−0.025	0.104	0.056	−0.049
	(0.035)	(0.036)	(0.029)	(0.039)		(0.076)	(0.108)	(0.095)	(0.129)
Fast Food Restaurants **^a,c^**									
1–4 Restaurants	0.006	−0.013	−0.021	0.103 *		0.044	−0.073	0.081	−0.330 *
	(0.035)	(0.043)	(0.039)	(0.047)		(0.109)	(0.219)	(0.168)	(0.161)
5–11 Restaurants	0.033	−0.069	0.016	0.049		0.001	−0.236	0.214	−0.151
	(0.053)	(0.055)	(0.049)	(0.060)		(0.130)	(0.265)	(0.217)	(0.206)
12 + Restaurants	0.065	−0.154 *	−0.029	0.098		−0.083	−0.133	0.095	0.044
	(0.064)	(0.063)	(0.051)	(0.068)		(0.154)	(0.280)	(0.225)	(0.228)
Convenience Stores ^a,c^									
1–2 Stores	0.084 *	−0.038	0.051	−0.059		−0.042	−0.006	−0.123	−0.118
	(0.035)	(0.040)	(0.037)	(0.048)		(0.103)	(0.174)	(0.154)	(0.200)
3–5 Stores	0.071	−0.002	0.027	−0.022		0.055	0.332	−0.432	−0.051
	(0.043)	(0.047)	(0.044)	(0.057)		(0.118)	(0.256)	(0.281)	(0.186)
6 + Stores	0.107	−0.009	−0.003	−0.019		−0.063	0.237	−0.377	−0.142
	(0.056)	(0.056)	(0.051)	(0.066)		(0.148)	(0.260)	(0.244)	(0.233)

**^a^** Difference-in difference estimate (b) with standard error (SE) of the effect of MOVE! on BMI change for “stayers,” or those whose home location did not move more than 0.25 mile from its location at baseline, from OLS regression models with person and calendar time fixed effects and the following covariates: marital status, 10 chronic health conditions, census tract median household income, census tract poverty rate, census tract population density, census division, urbanicity, VA facility, distance to the nearest VA facilities for outpatient and inpatient care, number of days from each target follow-up date and the actual weight measurement date, grocery stores, parks, and fitness facilities; **^b^** MOVE! effect for those with no supermarket, fast food restaurant, or convenience store within a 1-mile buffer; **^c^** Difference in MOVE! effect on BMI change relative to those with no food outlets. * *p* < 0.05; ** *p* < 0.01; *** *p* < 0.001.

## Supplementary Materials

The following are available online at http://www.mdpi.com/1660-4601/15/2/211/s1, Table S1: MOVE! effect on BMI at 6, 12, 18, and 24 months by food outlet access for (a) patients with complete data (all timepoints) and (b) patients with at least baseline and 18 month observations, Table S2: MOVE! effect on BMI at 6, 12, 18, and 24 months by food outlet access, including supermarket and grocery stores combined, Table S3: MOVE! effect on BMI at 6, 12, 18, and 24 months by food outlet access, including large chain supermarkets, Table S4: MOVE! effect on BMI at 6, 12, 18, and 24 months by food outlet access, including chain and non-chain fast food restaurants, Table S5: MOVE! effect on BMI at 6, 12, 18, and 24 months by food outlet access, including relative accessibility of supermarkets to fast food restaurants, Table S6: MOVE! effect on BMI at 6, 12, 18, and 24 months by food outlet access at 3 miles, Table S7: MOVE! effect on BMI at 6, 12, 18, and 24 months by food outlet access at 0.5 miles, Table S8: MOVE! effect on BMI at 6, 12, 18, and 24 months by distance (in miles) to the nearest food outlet.
